# An Unpleasant Surprise: Left Ventricular Pseudoaneurysm Developing After Placement in Trendelenburg Position

**DOI:** 10.7759/cureus.10245

**Published:** 2020-09-04

**Authors:** Nicholas T Manasewitsch, Daniel Antwi-Amoabeng, Eric Lu, Bryce D Beutler, Christopher J Rowan

**Affiliations:** 1 Internal Medicine, University of Nevada, Reno School of Medicine, Reno, USA; 2 Cardiology, University of Nevada, Reno School of Medicine, Reno, USA

**Keywords:** free wall rupture, false aneurysm, left ventricular pseudoaneurysm, myocardial infarction, trendelenburg

## Abstract

Free wall rupture after a myocardial infarction may rarely cause a left ventricular (LV) pseudoaneurysm to develop. LV pseudoaneurysms are most commonly discovered incidentally on echocardiography and require a high index of suspicion to diagnose. We report the case of a 73-year-old male who experienced an asymptomatic myocardial infarction leading to cardiac arrest after placement in the Trendelenburg position. During resuscitation efforts, he was discovered to have an LV pseudoaneurysm on transthoracic echocardiogram. We report an unusual presentation of LV pseudoaneurysm and discuss a possible link between Trendelenburg position and the development of LV pseudoaneurysm.

## Introduction

Left ventricular (LV) pseudoaneurysm is a rare complication of myocardial infarction (MI) that is difficult to diagnose given its nonspecific presentation. A majority of LV pseudoaneurysms are discovered incidentally on echocardiography, and many presenting symptoms are neither sensitive nor specific for an LV pseudoaneurysm [1-3]. Approximately 12%-48% of patients can be completely asymptomatic at presentation [3,4]. Therefore, the clinician must maintain a high index of suspicion. The most common causes of LV pseudoaneurysms are MI (55%), surgery (33%), trauma (7%), and infection (5%). Patients can present with heart failure, chest pain, and dyspnea [4]. These findings are usually already present in many patients with coronary artery disease illustrating a diagnostic challenge. We present the case of a 73-year-old male who experienced a recent asymptomatic MI and was discovered to have an LV pseudoaneurysm by transthoracic two-dimensional echocardiography after initial cardiac resuscitation.

## Case presentation

A 73-year-old male presented to the emergency department with altered mental status following a syncopal event. He had a past medical history of coronary artery disease with cardiac bypass 16 years prior, diabetes, hyperlipidemia, and Alzheimer's dementia. According to his wife, the patient had been feeling poorly with decreased appetite for several days and had complained of left neck pain the night prior to presenting to a local health center. He was transferred to our tertiary facility on suspicion of diabetic ketoacidosis or sepsis.

**Figure 1:**
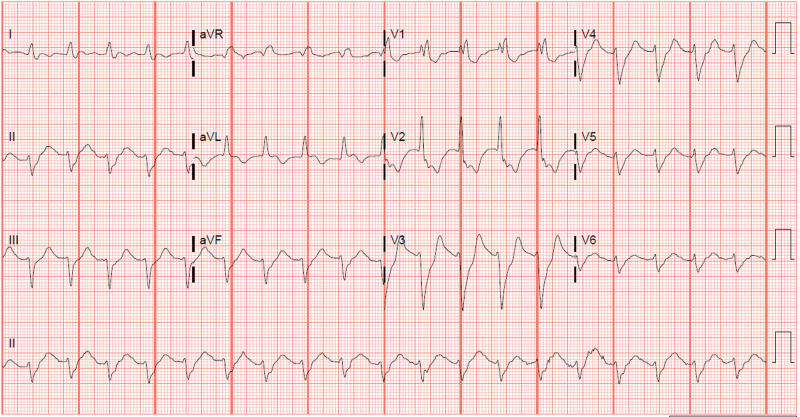
Electrocardiogram (ECG) showing supraventricular tachycardia with aberrant conduction secondary to right bundle branch block, a right atrial abnormality, and a left anterior fascicular block without ST, T-wave, or Q-wave changes.

He was ill appearing with the Kussmaul breathing pattern and mottled skin. His vital signs were blood pressure of 94/61 mmHg, respiratory rate of 24 breaths/minute, heart rate of 110 beats/minute, and SpO_2_ 97%. Initial laboratory workup showed white blood cell count of 21.5 K/µL (reference range: 4.8-10.8 K/µL), high anion gap metabolic acidosis with bicarbonate of 10 mEq/L (reference range: 20-33 mEq/L), anion gap of 30 mEq/L (reference range: 7-16 mEq/L), serum lactic acid level of 13.8 mEq/L (reference range: 0.5-2 mEq/L), ultra-high sensitivity (Gen 5) troponin T at 3,695 ng/L (reference range: 6-19 ng/L), and glucose of 501 mg/dL (reference range: 65-99 mg/dL). Electrocardiogram (ECG) (Figure 1) exhibited supraventricular tachycardia (SVT) with aberrant conduction secondary to right bundle branch block, a right atrial abnormality, and a left anterior fascicular block without ST, T wave, or Q wave changes. A CT scan of the head without contrast showed no signs of an acute process.

**Figure 2:**
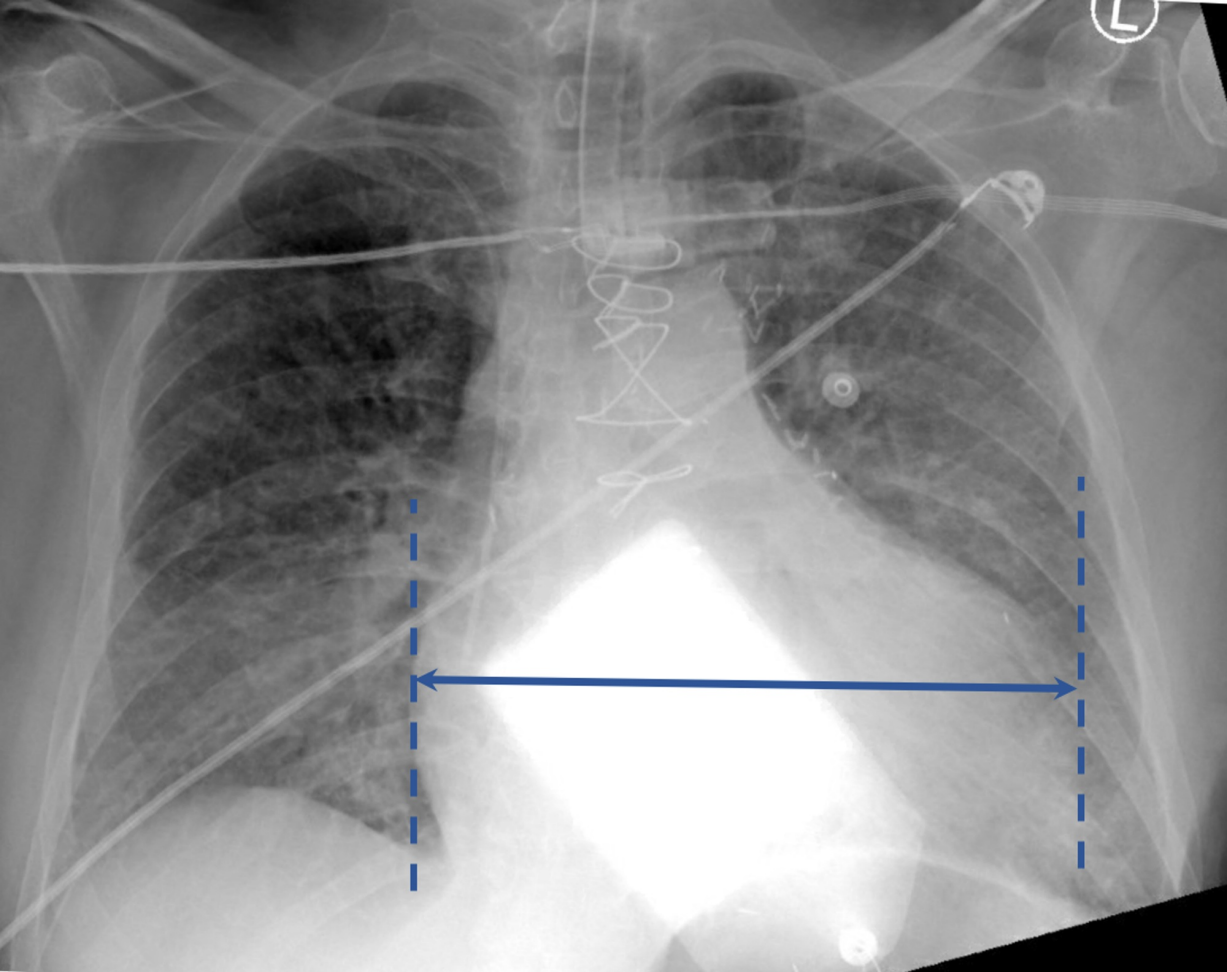
Portable chest x-ray demonstrating enlarged cardiac silhouette (arrow), pulmonary edema, and midline sternotomy wires.

A portable chest x-ray (Figure 2) showed an enlarged cardiac silhouette, pulmonary edema, and median sternotomy wires from his previous cardiac bypass. Arterial blood gas elicited pH of 7.015 (reference range: 7.4-7.5), temperature-corrected PCO_2_ of 22.6 mmHg (reference range: 26-37 mmHg), and PO_2_ of 84 mmHg (reference range: 64-87 mmHg). 

**Figure 3:**
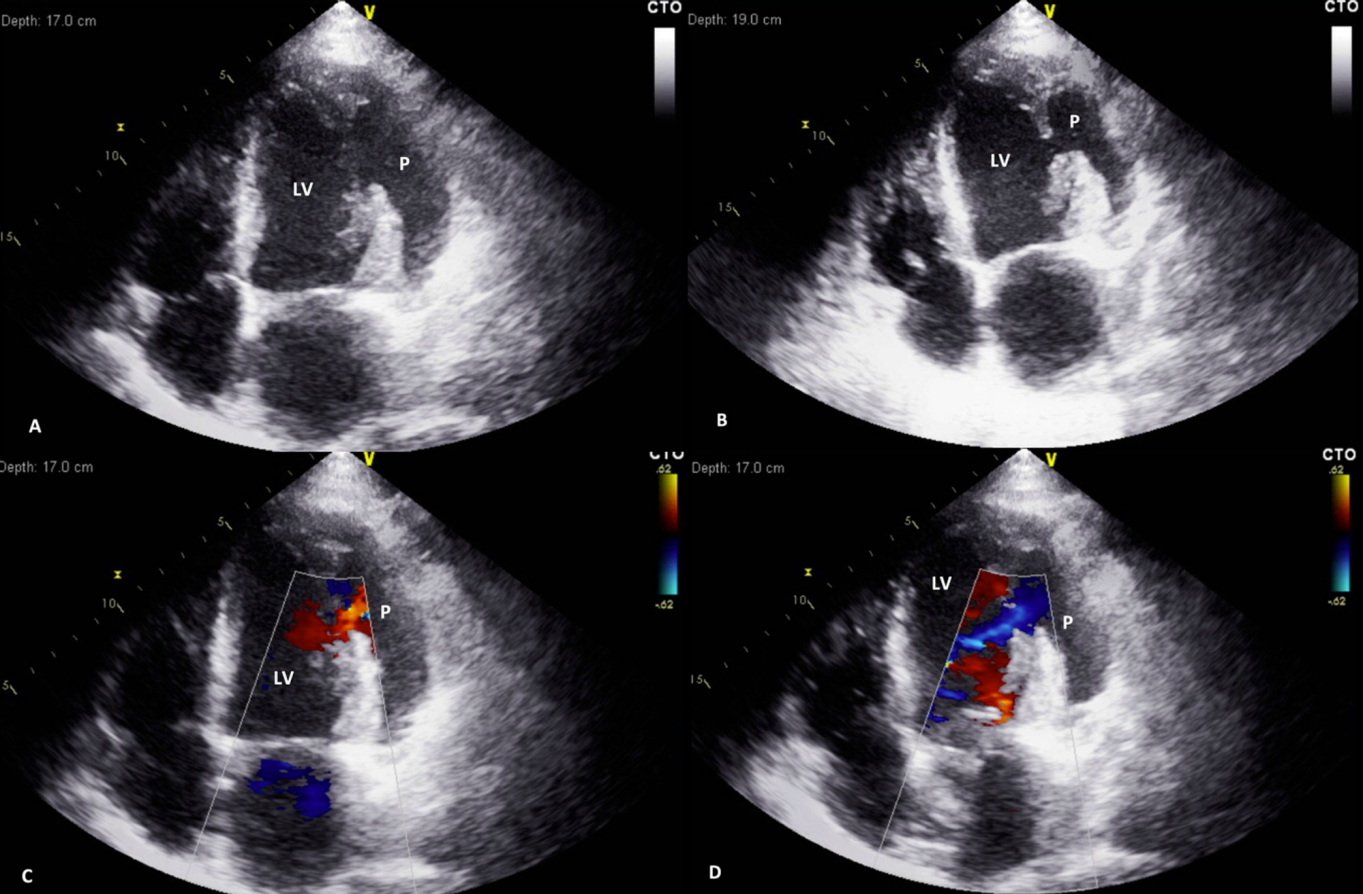
Apical four-chamber views of transthoracic echocardiography demonstrate LV pseudoaneurysm of the lateral wall, severely reduced LV systolic function (<20%), and normal pericardium without effusion (A and B). Color Doppler echocardiography indicates bidirectional flow across a narrow orifice that communicates between the left ventricle and the pseudoaneurysm (C and D). LV = left ventricle; P = pseudoaneurysm

The patient was resuscitated with multiple boluses of fluids, treated with insulin, and sedated. While further workup and treatment was underway, the patient was placed in Trendelenburg position for a central line placement and within two minutes developed asystolic cardiac arrest. Initially, he was successfully resuscitated with return of spontaneous circulation (ROSC) after five minutes. He was placed on full mechanical ventilatory support with central venous catheter and vasopressor therapy. More than an hour after the initial asystolic cardiac arrest and as the patient continued to experience two recurrent episodes of cardiac arrest, a bedside transthoracic two-dimensional echocardiogram was performed demonstrating a large LV pseudoaneurysm of the lateral wall and severely reduced LV ejection function <20% (Figure 3).

Unfortunately, the patient continued to have subsequent episodes of asystolic cardiac arrests for over two hours after receiving standard resuscitation treatment per the Advanced Cardiac Life Support protocol. Cardiology was consulted and concluded that cardiac catheterization would have limited utility. Given our patient’s two recurrent, extended asystolic cardiac arrest now in the context of his large LV pseudoaneurysm, he was transitioned to comfort care and passed shortly thereafter.

## Discussion

Free wall rupture is a devastating consequence of an ischemic cardiac event, occurring in 4% of patients after an MI [5]. LV pseudoaneurysms are very rare complications with an estimated incidence of 0.23% [6]. The pathophysiology involves a free wall rupture that is contained by adherent overlying pericardium and scar tissue that prevents development of hemopericardium and tamponade. LV pseudoaneurysms often have a narrow orifice that is usually <50% the diameter of the pseudoaneurysm [7]. This differs from an LV true aneurysm that forms due to weakening of the myocardial wall, which causes outward bulging of the LV with a broad, rather than narrow, base.

Abnormal chest X-ray findings (97%) and abnormal ECG findings (95%) are near universal in LV pseudoaneurysm [4]. The most common chest X-ray finding is an enlarged cardiac silhouette, and the most commonly discovered ECG findings are nonspecific ST changes (74%), anterior ST elevation (12%), and posterior ST elevation (9%). In a retrospective review of 10 patients with LV pseudoaneurysm, all 10 patients had ECG abnormalities, such as T-wave changes, nonspecific Q waves, or nonspecific ST changes [8]. Even when presenting with ST elevations, LV aneurysms were the most frequently misdiagnosed ST elevation pattern among emergency medicine physicians [9].

Transthoracic echocardiography is useful in detecting many cases of LV pseudoaneurysm and is often the first diagnostic test of choice although further imaging studies such as angiography, transesophageal echocardiogram, cardiac CT, and cardiac MRI are helpful [4,7]. Two-dimensional and three-dimensional echocardiography with Doppler flow can be highly diagnostic [7]. Characteristic findings on two-dimensional echocardiography are a sharp discontinuation of the endocardium, an echo-free saccular or globular space, and a narrow orifice connection at the site of the LV pseudoaneurysm [10]. Cardiac MRI is an emerging and promising imaging modality for diagnosis, particularly in patients with uncertain echocardiography findings [11]. LV pseudoaneurysms most commonly present on the posterior, lateral, apical, or inferior surfaces of the LV with a minority presenting on the anterior or basal surfaces [4]. It is proposed that anterior LV pseudoaneurysms are less common because these are more likely to rupture and cause tamponade, shock, and death compared to posterior pseudoaneurysms [1,4].

In our case, the patient’s symptoms and ECG findings were not suggestive of an LV pseudoaneurysm. The decision to perform a bedside transthoracic echocardiogram after initial ROSC was crucial to determining the patient’s diagnosis and confirming his poor prognosis. Our patient’s two-dimensional echocardiography study demonstrated severely reduced LV systolic function <20% and a normal pericardium without effusion (Figure 3). The color Doppler ultrasonography illustrated bidirectional flow across a narrow orifice that communicates between the LV and the pseudoaneurysm.

The main goal of treatment is to decrease the probability of rupture and prevent tamponade, shock, and death. Additionally, a dyskinetic or akinetic myocardium due to LV pseudoaneurysm can lead to arrhythmia and cardiac failure, while blood stasis increases the risk of thrombotic embolism [1]. There is currently no standard of treatment or imaging follow-up for pseudoaneurysms given its low incidence though in general, surgical treatment has better prognosis compared to medical management. Medical management has more than double the mortality rate of surgery (48% vs 23%, respectively); thus, prompt diagnosis of LV pseudoaneurysm and urgent surgical evaluation is imperative [1,4]. Surgical mortality of LV pseudoaneurysm has been reported to be as low as 7% [3].

Our case is unique because our patient had an ECG that did not initially suggest an MI or an LV pseudoaneurysm. As mentioned previously, ECG findings are present in 95% of patients with LV pseudoaneurysm [4]. Our patient’s elevated troponins indicate that our patient likely had an MI in his recent past. Additionally, the LV pseudoaneurysm was not discovered until after the patient’s first cardiac arrest minutes after he was placed in the Trendelenburg position. We propose two possible explanations for this series of events: (1) our patient was among the 5% of LV pseudoaneurysms that did not present with ECG findings and (2) our patient developed the LV pseudoaneurysm after the ECG was performed and after he was placed in the Trendelenburg position.

With the second possible explanation, we propose that our patient had a recent MI which weakened his myocardium, and the Trendelenburg position increased LV preload which triggered the free wall rupture and LV pseudoaneurysm formation. A recent, asymptomatic MI was probable in our patient given his past medical history of coronary artery disease, hyperlipidemia, and diabetes as well as elevated troponin on presentation. In a systematic review of the Trendelenburg position and its effect on hemodynamics, Ballesteros-Peña et al concluded that Trendelenburg position increases LV preload and cardiac output [12]. Thus, our patient may have experienced a rapid increase in his preload that increased pressure on the already weakened myocardial wall, leading to LV pseudoaneurysm formation. The fact that the patient did not initially have ECG findings that would typically appear with LV pseudoaneurysm supports this notion, and the timing of the patient’s cardiac arrest within two minutes of being placed in Trendelenburg position also support this hypothesis. To our knowledge, this is the first proposal of this link between Trendelenburg position and LV pseudoaneurysm formation in the current literature.

Combined, these characteristics highlight the unusual presentation of an already rare disease process and further support the current literature that LV pseudoaneurysms remain a diagnostic challenge. This case demonstrates the difficult and sometimes unusual presentation of LV pseudoaneurysm in a patient without a history suggestive of an MI.

## Conclusions

LV pseudoaneurysms are most commonly discovered incidentally on echocardiography and require a high index of suspicion to diagnose. We report the case of LV pseudoaneurysm presenting as altered mental status leading to cardiac arrest which presented following an asymptomatic MI with no related ECG changes. We believe that LV pseudoaneurysms require a high index of suspicion and that advances in imaging will enable more prompt diagnosis and urgent surgical treatment.
